# The Effect of Soil-Climate Conditions, Farmyard Manure and Mineral Fertilizers on Potato Yield and Soil Chemical Parameters

**DOI:** 10.3390/plants10112473

**Published:** 2021-11-16

**Authors:** Lukáš Hlisnikovský, Ladislav Menšík, Eva Kunzová

**Affiliations:** Division of Crop Management Systems, Crop Research Institute, Drnovská 507/73, 161 06 Prague, Czech Republic; ladislav.mensik@vurv.cz (L.M.); kunzova@vurv.cz (E.K.)

**Keywords:** *Solanum tuberosum* L., cattle manure, mineral N, P and K, weather conditions, soil pH, soil nutrient content, PCA, FA

## Abstract

If available to farmers, potatoes represent a crop classically fertilized with farmyard manure in the Czech Republic. At the same time, potatoes are a crop sensitive to soil–climate conditions. We evaluated the effect of cattle manure (FYM), manure and mineral nitrogen (FYM + N1, FYM + N2), manure and mineral fertilizers (FYM + N1PK, FYM + N2PK, FYM + N3PK) application and the effect of three soil-climatic conditions (Caslav—maize production area with degraded Chernozem, Ivanovice—maize production area with Chernozem, Lukavec—potatoes production area with Cambisol) over four years (2016–2019) on potatoes yield and soil chemical properties. Of all the factors, yields were most affected by location. Lukavec provided the highest average yields (37.2 t ha^−1^), followed by Ivanovice (23.5 t ha^−1^) and Caslav (15.5 t ha^−1^). The second most important factor was the climatic conditions of the year. Fertilization was the third most important parameter. FYM significantly increased yields compared to Control, but applied alone cannot cover the needs of potatoes. Similarly, the application of FYM and N increases yields, but for the highest yields, it is best to apply FYM + NPK (80 kg ha^−1^ N). Co-application of FYM and mineral N fertilizers mitigates the negative impact of mineral N on soil pH.

## 1. Introduction

**Figure 1:**
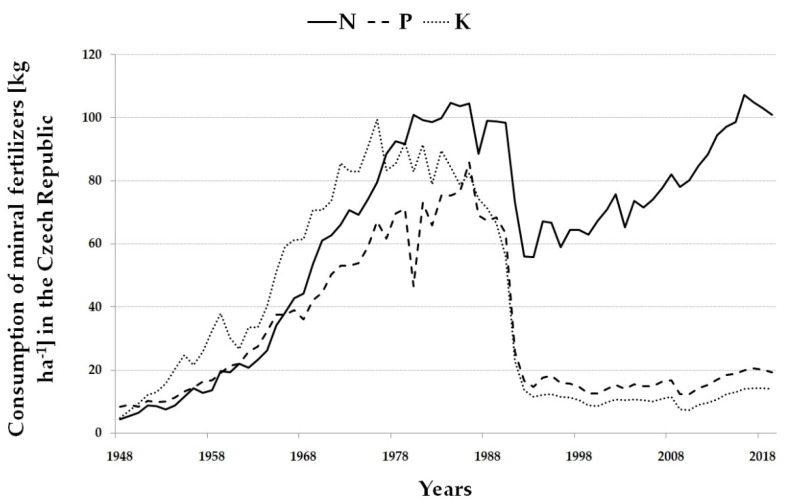
Average consumption of mineral N, P and K (kg ha^−1^) in the Czech Republic (1948–2019).

Fertilizer application is the cornerstone of crop production. The origins of fertilization are linked to the Neolithic Revolution when people switched from hunting and gathering to agriculture. People began to settle at the expense of migration, built their first settlements and started to collect various forms of waste in pits located near their houses. Such pits are documented in Sumerian cities, in the period around 6000 BC [1]. More recent research has shown that even in earlier times people used manure and water management to increase crop yields [2]. Even today, organic manures are an essential element of crop production, together with organic and mineral fertilizers. All three groups of fertilizers (organic manures, organic and mineral fertilizers) are characterized by different mechanisms of action on soil and crops. Organic manures have a beneficial effect on the physical, chemical and biological parameters of the soil [3,4,5,6,7,8], but their nutrient content is relatively low and must therefore be applied in large doses. The composition of individual organic manures is not homogeneous, varying both within and between types (slurries, manures), depending on their origin [9]. The rate of mineralization of manure strongly depends on the type of manure and the climatic conditions. While organic manures with a low C:N ratio (slurries) provide the most nutrients in the first year of application, manures with a high C:N ratio (farmyard manures—FYM) release nutrients to a lesser extent but over a longer period [10]. However, even organic manures can harm the environment, either through over-fertilization or through the presence of undesirable substances that enter the soil and water through the application, such as veterinary pharmaceuticals [11]. Mineral fertilizers have a homogeneous and precisely known composition and their effect is rapid. Mineral fertilizers are behind the success of conventional agriculture and ensure the production of basic raw materials for a wide range of industries. On the other hand, mineral fertilizers represent the source of environmental pollution [12,13,14,15], which is one of the aspects negatively influencing the public’s view on conventional agriculture and is behind the growing interest in organic farming [16]. Agriculture in the Czech Republic is characterized by an imbalance between livestock and crop production due to the changes in the crop rotations (reduction of perennial fodder crops and cereals in favor of winter rapeseed), reduced animal husbandry (there are areas completely devoid of livestock production), which leads to the low organic manure inputs, and finally by the imbalance between applied mineral nutrients [17] (high application of nitrogen fertilizers, low application of phosphate and potassium fertilizers, Figure 1, and low level of liming [18]). A similar trend for mineral fertilizers can be discerned in neighboring Poland [19]. All these aspects lead to soil degradation, which has been evident in the Czech Republic for a long time [20].

While the application of mineral nitrogen and potassium can satisfy crop requirements, it is also associated with negative aspects such as pH reduction and depletion of other nutrients [21,22,23]. And as the soil pH decreases, the mobility of risk elements (heavy metals, such as Cd, Cu, Mn, Pb and Zn) in the soil increases [15], which can negatively affect the quality of crop production [24].

The yield and quality of crops are not only affected by fertilization and management practices. Soil and climatic conditions also play an important role. Potatoes are one of the most nutrients and moisture demanding crops, requiring high amounts of N due to the poor N efficiency [25]. They tend to prefer lighter soil types, which are found at higher altitudes, receiving higher rainfall, which compensates for the negative aspect of light soils—drying out. Comparing the effect of climatic conditions, both temperature and precipitation play an important role in yield forming. This is especially important nowadays when we are exposed to changing climatic conditions and more frequent occurrences of unusual (extreme or extraordinary) phenomena [26,27]. High temperatures can negatively affect the efficiency of photosynthesis, water management and respiration. However, the temperature seems to play a less important role as a factor affecting potatoes yields than precipitation, which is a more important yield formatting—factor and can compensate for the negative effects of high temperatures [28] and increase fertilizer utilization [29]. The precipitation and soil type are so important factors for potatoes that even the most naturally fertile soils (Chernozems) in the Czech Republic [30] cannot provide high potato yields without the proper climate conditions. In other words, it means that naturally created barriers strongly limit the farmer’s options, regardless of fertilization or farming practices.

In the Czech Republic, potatoes are traditionally fertilized with FYM (if available) in the first line. Manure not only gradually adds nutrients, but in heavy (clay) soils, which are not conducive to good potato growth, it acts as an aerating agent and alleviates soil’s heaviness. On the other hand, in light (sandy) soils, manure provides organic matter and nutrients that would otherwise be lacking. Application of manure significantly increases the potatoes yield [31] and also affects yields and soil chemical composition a long time after the manure application [32]. However, organic manures (such as FYM) cannot supply enough nutrients to meet the needs and potential of modern potato varieties. For this reason, it is advisable to apply mineral fertilizers [25,33] or combine organic manures with mineral fertilizers [34]. However, fertilization recommendations cannot be generalized, as each recommendation should be site-specific, based on the soil and climate conditions of the site [35].

Our main research goal was to assess characteristics of the interactions between differentiated fertilization management (seven fertilization treatments) and environmental factors in aspects of its influence on the potato yields and selected soil parameters (pH, N, P, K and soil carbon content—Cox). The fertilization treatments represent different management practices and include 1) unfertilized Control, 2) application of cow manure (FYM), 3, 4) combination of manure and two different mineral nitrogen rates (FYM + N1, FYM + N2), which represents the direction of fertilization without the application of mineral P and K fertilizers, and 5, 6 and 7) the combination of FYM and mineral NPK fertilizers (FYM + N1PK, FYM + N2PK, FYM + N3PK), which represents the combination of manure and all three major mineral fertilizers (against FYM + N treatments). The experiment was conducted between the years 2016 and 2019 (four years) on three sites with different soil and climatic conditions (Caslav—degraded Chernozem, Ivanovice—Chernozem, Lukavec—Cambisol).

## 2. Results

### 2.1. Weather Conditions

#### 2.1.1. Caslav

In Caslav, the weather conditions were the main factor influencing yields (see Section 2.2.1, 67% according to the MANOVA). The lowest average yields were recorded in 2018 (7.7 t ha^−1^, Table 1), which was the season characterised as a season with precipitation very below normal (Table S1). The sum of precipitation was very below normal during April and May and extraordinary below normal during July (Table S1). The year 2018 was also the hottest one. April and August were especially hot, characterized as extraordinary above normal (Table S2), and the whole season was very above normal. This means that 2018 was a very dry and warm year in Caslav, which affected the yield.

#### 2.1.2. Ivanovice

A similar situation was recorded in Ivanovice. Yields here were largely influenced by weather conditions (see Section 2.2.2, 87% according to the MANOVA). The lowest average yields were recorded in 2018 (12.0 t ha^−1^, Table 1). The 2018 season was characterized as the season with the lowest sum of precipitation (228.5 mm during the season, Table S1). The 2018 season was also the hottest one. With the average temperature of 18.8°C, the 2018 season was extraordinary above the normal season, with two months (April and August) being extraordinary above normal (Table S2). As in Caslav, the combination of unprecedented conditions in 2018 resulted in extraordinary low yields in 2018.

#### 2.1.3. Lukavec

In Lukavec, the lowest average yields were recorded in 2019 (26.3 t ha^−1^) and 2018 (30.2 t ha^−1^, Table 1). In both seasons we recorded extraordinary above normal temperatures (April and August in 2018 and June in 2019, Table S2). Also, April (the month of planting) was dry in both years, in 2019 followed by very cold May and extraordinary hot June (Table S1), which negatively affected plant development, resulting in the lowest yields in this season.

Taking a closer look at the effect of precipitation and temperature at each site, the temperature was always strongly and negatively correlated with the yield at all three sites (Caslav–r = −0.76, Ivanovice–r = −0.86, Lukavec–r = −0.62), while precipitation was positively correlated, very weakly at Caslav (r = 0.25), moderately at Ivanovice (r = 0.65) and at Ivanovice (r = 0.75).

### 2.2. Potato Yields

The potato yields were significantly affected by locality (d.f. = 2, F = 1412, *p* < 0.001, the factor “locality” affected the potato yields by 66%), year (d.f. = 3, F = 359, *p* < 0.001, 17%), fertilizer treatment (d.f. = 6, F = 192, *p* < 0.001, 9%), locality × year interaction (d.f. = 6, F = 162, *p* < 0.001, 7%), followed by the fertilizer treatment × year and locality × fertilizer treatment × year interaction (0.3% together). Thus, the results show that potato yields were most influenced by the location of cultivation, then by the factor year, followed by fertilization. The lowest average yields were harvested in Caslav (15.5 t ha^−1^), followed by Ivanovice (23.5 t ha^−1^) and Lukavec (37.2 t ha^−1^). All three results are statistically significantly different. The lowest average yields were recorded in 2018 (16.6 t ha^−1^), followed by 2019 (25.0 t ha^−1^), 2017 (28.9 t ha^−1^), and 2016 (31.2 t ha^−1^). All four results are statistically significantly different. Fertilization treatments provided average yields in ascending order: Control (14.9 t ha^−1^), FYM (20.8 t ha^−1^), FYM + N1 (20.8 t ha^−1^), FYM + N2 (26.1 t ha^−1^), FYM + NPK1 (28.5 t ha^−1^), FYM + NPK2 (31.2 t ha^−1^), FYM + NPK3 (32.6 t ha^−1^). From Control to FYM + NPK1 treatments, all results are statistically significantly different. Yields provided by FYM + NPK2 and FYM + NPK3 are statistically insignificant.

#### 2.2.1. Caslav

In Caslav, the potato yields were significantly affected by the year (d.f. = 3, F = 421, *p* < 0.001, 67%), followed by the fertilizer treatment (d.f. = 6, F = 191, *p* < 0.001, 30%) and year × fertilizer treatment interaction (d.f. = 18, F = 17, *p* < 0.001, 3%). The lowest mean yields were harvested in 2018 (7.7 t ha^−1^), while the highest harvest was recorded in 2016 (21.7 t ha^−1^). Each year results are statistically different (Table 1). Comparing the fertilizer treatments, the mean yields ranged from 7.7 (Control) to 22.1 (FYM + NPK3) t ha^−1^ (Table 1). Application of FYM significantly increased the potato yields when compared with the unfertilized Control (+2.9 t ha^−1^). The application of FYM with mineral N treatments (FYM + N1, FYM + N2) slightly (and significantly) increased the yields when compared with the FYM (Table 1). The addition of PK fertilizers resulted in significantly higher yields when compared with FYM + N treatments. Finally, no difference was found between the FYM + NPK2 and FYM + NPK3 treatments (Table 1), showing that the dose of 80 kg ha^−1^ N applied together with mineral PK fertilizers results in high yields and is optimal (application of 120 kg ha^−1^ N is not necessary, the potato yields are not significantly different).

#### 2.2.2. Ivanovice

In Ivanovice, the potato yields were significantly affected by the year (d.f. = 3, F = 178, *p* < 0.001, 87%), fertilizer treatment (d.f. = 6, F = 25, *p* < 0.001, 12%), and the year × fertilizer treatment interaction (d.f. = 18, F = 2, *p* < 0.001, 2%). The lowest mean yields were harvested, as in Caslav, in 2018 (12.0 t ha^−1^), due to the poor weather conditions. The highest yields were recorded in 2016 (29.9 t ha^−1^) and 2019 (30.3 t ha^−1^, Table 1). If we compare the fertilization treatments, we can see that the trend is very similar to Caslav, i.e., yields increase with increasing nutrient inputs. In the case of Ivanovice, however, the differences between the treatments are not so sharp (are overlapping) and all three FYM + NPK treatments provided comparable results. Moreover, the application of FYM resulted in yields comparable to those of all treatments up to FYM + NPK1 (Table 1). The explanation lies in the soil and climatic conditions. In terms of climate, both sites (Caslav and Ivanovice) are comparable. In terms of soil, in Caslav, the crops are grown on degraded chernozem, which is a soil poorer in nutrients (compared to chernozem in Ivanovice) and the crops respond very well and willingly to the nutrients supplied. In Ivanovice the soil is naturally fertile, it is one of the best soils in the Czech Republic, therefore the response of potatoes to the supplied nutrients (via fertilizers) is not so significant and the application of FYM is sufficient to obtain a satisfactory harvest (the yield difference between FYM and FYM + NPK1 is only 3.5 t ha^−1^ and the difference is insignificant).

#### 2.2.3. Lukavec

In Lukavec, the potato yields were significantly affected by the year (d.f. = 3, F = 236, *p* < 0.001, 70%), fertilizer treatment (d.f. = 6, F = 91, *p* < 0.001, 27%) and year × fertilizer treatment interaction (d.f. = 18, F = 8, *p* < 0.001, 3%). The lowest potato yields were recorded in 2019 (26.3 t ha^−1^), while the highest average yields were harvested in 2017 (50.6 t ha^−1^, Table 1). As in the previous two sites, average yields increased with the dosage of nutrients supplied (Table 1). Unfertilized Control provided the lowest average yields (22.0 t ha^−1^), while the highest yields were harvested when FYM + NPK3 was applied (47.9 t ha^−1^). The FYM + NPK2 treatment (45.2 t ha^−1^) provided comparable potato yields to the FYM + NPK3 treatment, the difference between the two treatments was 2.7 t ha^−1^ and was not statistically significant (Table 1). The application of 80 kg of mineral N, together with PK fertilizers (FYM + NPK2) is sufficient to achieve decent yields and a 50% increase in the dose of mineral N (FYM + NPK2—FYM NPK3) is not associated with a significant increase in yields.

### 2.3. Soil Properties

#### 2.3.1. Caslav

The average P concentration in Caslav ranged from 46 mg kg^−1^ (FYM) to 158 mg kg^−1^ (FYM + NPK2), Table 2. No statistically significant differences were observed for the treatments without mineral P (Control, FYM, FYM + N2) (Table 2). Thus, without mineral P application, soil P concentrations were low (Control, FYM) or suitable (FYM + N2). It must be said that the average concentration of 55 mg kg^−1^ (FYM + N2) is at the very lower end of the range for classification as “Suitable”. Thus, without mineral phosphate fertilizer application, the P concentration in the soil is poor and significantly affects potato yields (statistically significant difference between FYM + N and FYM + NPK treatments, Table 1).

A similar situation occurred in the case of K, with average soil K values ranging from 120 mg kg^−1^ (Control) to 221 mg kg^−1^ (FYM + NPK2). Again, the differences between Control, FYM and FYM + N2 were not significant (Table 2). In contrast, the application of mineral K fertilizers significantly increased soil K concentration to the “Good” level. Also, in the case of yields, we can see here a significant role (together with P) of mineral K, as the differences between treatments with and without mineral P and K fertilizers are significant (Table 1).

In the case of Mg and Ca, fertilizer application did not play a significant role and the differences between the measured concentrations were not statistically significant (Table 1). The average Mg concentration ranged from 136 mg kg^−1^ (FYM) to 164 mg kg^−1^ (FYM + NPK2). Mean Ca concentrations ranged from 2802 mg kg^−1^ (FYM + NPK2) to 3777 mg kg^−1^ (FYM).

Mean soil pH was not significantly different between fertilizer treatments and ranged from 6.51 (FYM + N2) to 6.85 (FYM). Similarly, the concentrations of Cox and Ntot were not significantly different between fertilization treatments and ranged from 1.17% (Control) to 1.29% (FYM + NPK2) for Cox and from 0.15 (Control) to 0.17% (FYM + NPK2) for Ntot (Table 3).

#### 2.3.2. Ivanovice

The P concentration in Ivanovice was significantly dependent on the fertilization treatment, with statistically different values in each fertilization treatment (Table 2). The lowest values were recorded in the unfertilized Control (66 mg kg^−1^, suitable), followed by FYM + N2 (117 mg kg^−1^, high), FYM (169 mg kg^−1^, high) and the highest concentration was in the FYM + NPK2 treatment (226 mg kg^−1^, very high).

The same situation occurred in the case of K. The lowest concentration was measured in Control (181 mg kg^−1^), followed by FYM + N2 (288 mg kg^−1^), FYM (370 mg kg^−1^) and FYM + NPK2 (447 mg kg^−1^). Statistically significant differences were recorded between the Control (A), FYM + N2 (B) and FYM (C) treatments together with FYM + NPK2 (C) (Table 2, fertilizer treatments followed by the same letter are statistically insignificant).

As in the previous case, fertilizer application had no significant effect on Mg and Ca concentrations, the differences were not statistically significant. The results of soil analyses for these two elements are shown in Table 2.

Soil pH was not affected by fertilization and ranged from 6.58 (Control) to 6.69 (FYM), Table 3. The Cox content was significantly affected by fertilization. The lowest concentration was measured in Control (1.67%), which was statistically significantly lower than in the other treatments, which were not significantly different from each other. Cox concentrations in these treatments ranged from 1.92% (FYM) to 2.07% (FYM + NPK2). In the case of Ntot, we did not record statistically significant differences between the treatments and ranged from 0.20% (Control) to 0.24% (FYM + NPK2), Table 3.

#### 2.3.3. Lukavec

In Lukavac, the lowest P concentration was recorded in the Control (44 mg kg^−1^), which was statistically comparable to the value of 46 mg kg^−1^ (FYM + N2). A higher P concentration was measured in the FYM treatment (90 mg kg^−1^), which provided a statistically significantly lower yield than FYM + N2 (Table 2), and thus we assume that P originating from FYM was not fully utilized in the case of the FYM treatment, resulting in a higher P concentration in the soil. The highest soil P concentrations were then observed in the FYM + NPK2 treatment, providing the second highest yields (Table 2), and the amount of P supplied was both sufficient to cover the requirements of the potato for high yield formation and sufficient to maintain high soil P levels.

The K concentrations varied significantly between fertilization treatments. The lowest concentration was recorded in Control (107 mg kg^−1^), followed by FYM + N2 (123 mg kg^−1^), FYM (147 mg kg^−1^) and FYM + NPK2 (167 mg kg^−1^).

In the case of Mg and Ca, the situation in Lukavac was similar to that in Caslav and Ivanovice; fertilizer application did not affect the concentration of these two elements in the soil. The results of soil analyses are shown in Table 2.

Soil pH was not significantly affected by fertilization treatments and ranged from 5.74 (FYM + N2) to 5.88 (FYM), Table 3. In the case of Cox, a significant difference was observed only between Control (1.41%) and FYM + NPK2 (1.82%). In the case of Ntot, the lowest concentration was measured in Control (0.19%), which was statistically significantly lower compared to the other fertilization treatments. For those, the concentration of Ntot was 0.23% (Table 3).

### 2.4. PCA and FA Results

**Figure 2:**
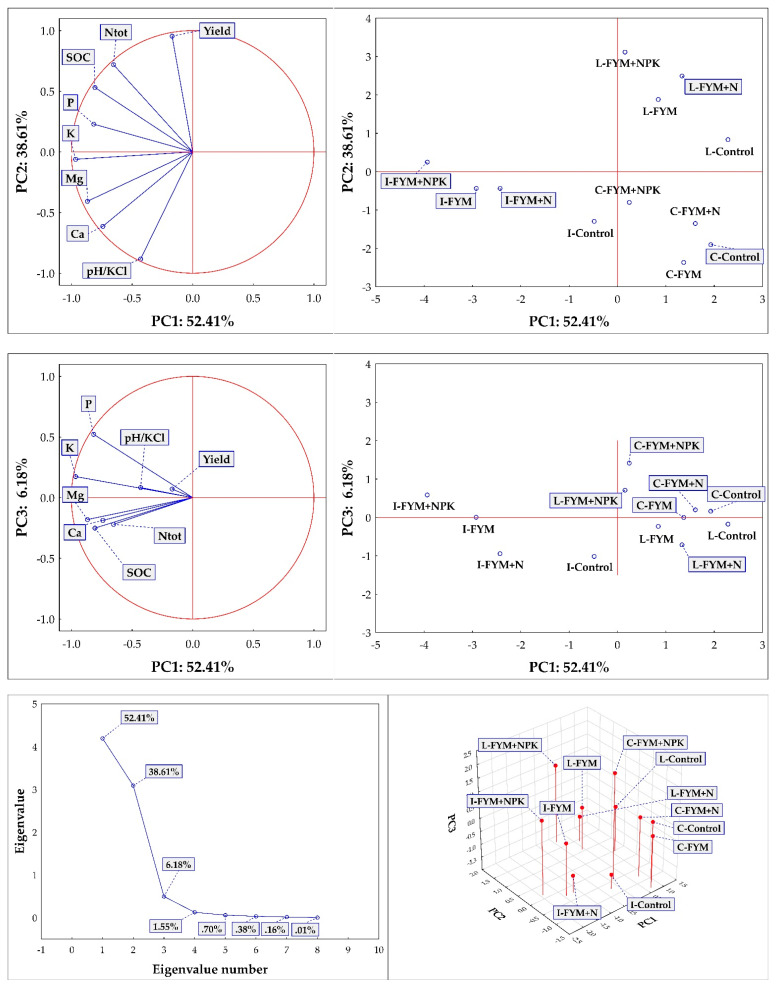
The results of the PCA analyses, including the eigenvalue of the parameters (Figure 2, left bottom side). Note: I—Ivanovice, C—Caslav, L—Lukavec, FYM—farmyard manure, FYM + N represents the FYM + N2 fertilizer treatment, FYM + NPK represents the FYM + NPK2 treatment, pH—soil reaction; P—phosphorus; K—potassium; Ca—calcium; Mg—magnesium; SOC—soil organic carbon—Cox; Nt—soil nitrogen content (Ntot).

In the plot of component weights PC1 and PC2 (Figure 2, top left) we can see that the first two axes are significant and together draw 91% of the variability. The PC1 axis in Figure 2, showing the relationship between PC1 and PC2 (Figure 2), characterizes the content of K, Mg and P, which are elements located in the plane with this axis and are strongly correlated with it (K–r = −0.96, Mg–r = −0.86, P–r = −0.81), as well as Cox, a parameter correlated at r = −0.80. Furthermore, there is a significant correlation with Ca on the PC1 axis (r = −0.74). There is a clearly significant correlation with yield (r = −0.95), pH (r = −0.88) and Ntot (r = 0.71) on the PC2 axis. There is no significant correlation on the PC3 axis.

In the component scatter diagram (Figure 2, top right), the sites (Caslav, Lukavec and Ivanovice) and fertilization treatments (C, FYM + N and FYM + NPK) are clearly located along the PC1 axis. The Ivanovice site is significantly to the left along the PC1 axis (highest available nutrient contents, highest Cox, Ntot and pH and lower yields compared to the other two sites—Lukavec and Caslav). The highest contents of available nutrients P and K and Cox and Nt contents, together with yields, were always recorded in the FYM + NPK treatment. And this is on all sites—the FYM + NPK treatment is always significantly more distinct within each cluster—site compared to the Control treatment, where we can find the lowest contents of available nutrients, Cox and Ntot. The FYM and FYM + N treatments at Lukavec and Ivanovice are very similar (clusters of variants close to each other). At the Caslav site, the Control, FYM and FYM+N treatments are very similar (a cluster of treatments close to each other), which is mainly due to the soil type at the site (degraded Chernozem). However, the differentiation within the PC2 axis is also significant. The Lukavec site is significantly different (Figure 1, upper right corner) from the other two sites (Ivanovice and Caslav). This is mainly due to two parameters—significantly lower pH (which suits the potatoes) and significantly higher yields. Lukavec represents a typical potato growing area, while Caslav and Ivanovice are mainly maize growing areas, more suitable for C4 crops.

**Figure 3:**
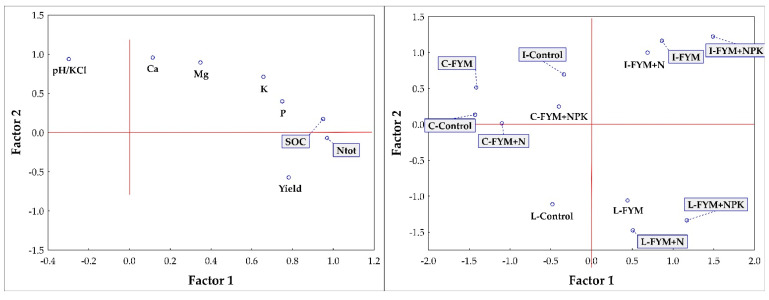
The FA (rotation: varimax normalized) of studied parameters (pH, Cox, Ntot, nutrients and yield) as affected by locality between the years 2016–2019. Note: pH—the value of the soil reaction; P—phosphorus; K—potassium; Ca—calcium; Mg—magnesium; SOC—soil organic carbon—Cox; Nt—soil nitrogen content (Ntot).

Factor analysis (FA, Figure 3) confirmed the PCA results and differentiated, similarly to PCA, groups of sites and fertilization treatments. Factor weights explain the correlations between factors and traits (Table 4). They represent the most important information on which the interpretation of the factors is based. It can be said that Factor 1 clearly describes Yield and also soil properties such as Cox, Ntot and additionally P content (significantly higher Cox, Ntot and P content in the FYM + NPK treatment compared to Control at all three sites and also the highest yields at Lukavec compared to Ivanovice and Caslav). Factor 2 clearly describes the content of accessible nutrients (Ca, Mg and K) and the pH value (significant differentiation of sites—the highest pH value, or Ca content, was always recorded in Ivanovice and Caslav, the lowest in Lukavec). Communality represents the proportion of trait variability expressed by the factors in question. The communalities are similar to the R2 value obtained when the original traits are explained by the regression of the selected factors [36]. The contribution of Factor 1 and Factor 2 to communality shows how communality takes high values (more than 0.9). Thus, the trait values are very well accounted for by the proposed factor model (Table 4).

### 2.5. Linear Regression Model Results

**Figure 4:**
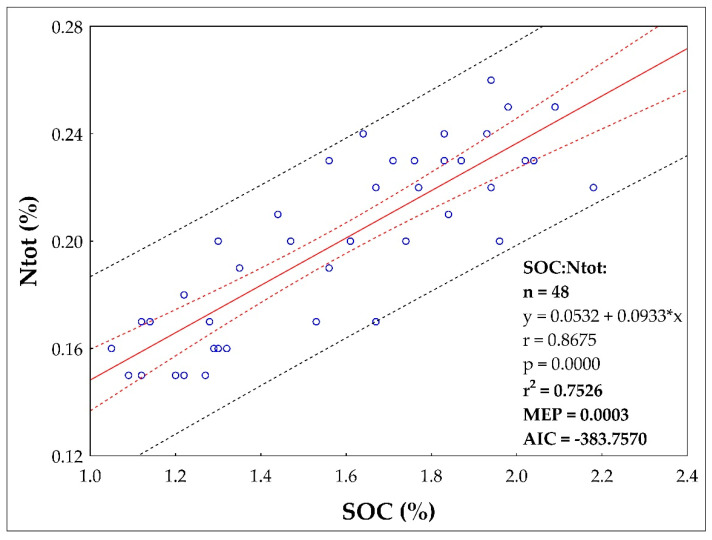
The regression linear relationship between the Cox (SOC) and Ntot between the years 2016 and 2019 (all three study sites—Caslav, Ivanovice, Lukavec).

The results of the soil analyses showed a linear regression relationship (data from all three sites) of Ntot content on Cox (Figure 4). The equation of the straight line relating the Ntot and Cox is estimated as: Ntot = (0.0532) + (0.0933) × Cox (using the 48 observations in the dataset). The statistical characteristics of the linear regression are as follows: r = 0.8675, R^2^ = 0.7526, MEP = 0.003, AIC = −383.7570. The linear regression model is significant according to Fisher–Snedecor test of model significance (F = 139.9522, quantile F = 4.0517, p = 1-4934E-015). The linear regression model is correct according to Scott’s multicollinearity criterion (SC = 0.2811). Residues show homoscedasticity (Cook–Weisberg test of heteroscedasticity). Residues have a normal distribution (according to the Jarque–Berr test of normality). Negative autocorrelation of residues was not demonstrated (according to the Durbin–Watson autocorrelation test). There is no apparent trend in the residuals. The y-intercept, the estimated value of Ntot when Cox is zero, is 0.0532 with a standard error of 0.0129. The slope, the estimated change in Ntot per unit change in Cox, is 0.0933 with a standard error of 0.0079. The estimated slope is 0.0933. The lower limit of the 95% confidence interval for the slope is 0.0774 and the upper limit is 0.1092. The estimated intercept is 0.0532. The lower limit of the 95% confidence interval for the intercept is 0.0273 and the upper limit is 0.0790.

**Figure 5:**
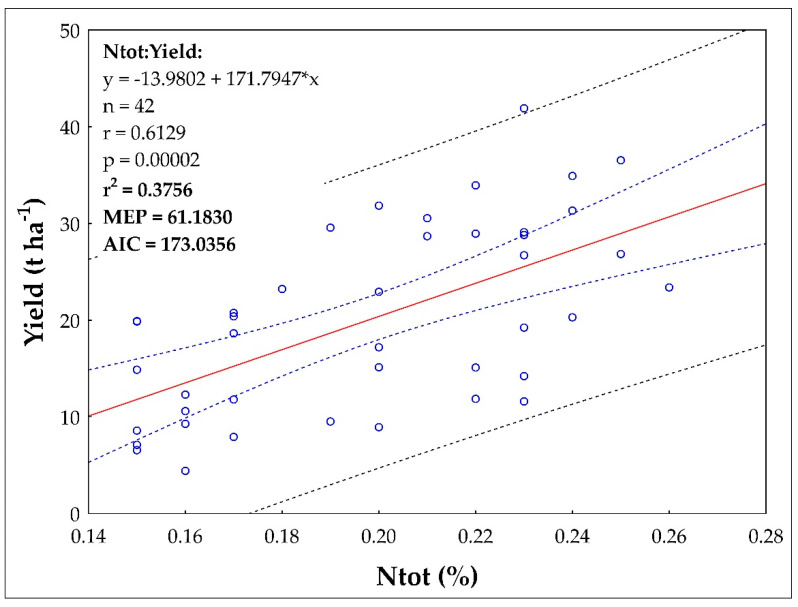
The regression linear relationship between the Ntot and Yield between the years 2016 and 2019 (all three study sites).

Furthermore, a statistically significant linear regression relationship (data from all 3 sites) of Yield on soil Ntot content was demonstrated (Figure 5). The equation of the straight-line relating Yield and Ntot is estimated as: Yield = (−13.9802) + (171.7947) × Ntot using the 42 observations in the dataset. The statistical characteristics of the regression are as follows: r = 0.6129, R^2^ = 0.3756, MEP = 61.1830, AIC = 173.0356. The model is significant according to the Fisher-Snedecor test of model significance (F = 24.0662, quantile F = 4.0847, *p* = 1.5981 × 10^−5^). The model is correct according to Scott’s multicollinearity criterion (SC = 0.2645). The residuals show homoscedasticity (Cook-Weisberg test for heteroscedasticity). The residuals have a normal distribution (Jarque-Berr normality test). Residuals are not autocorrelated (Durbin-Watson autocorrelation test). There is no trend in the residuals. The y-intercept, the estimated value of Yield when Ntot is zero, is −13.9802 with a standard error of 7.0537. The slope, the estimated change in Yield per unit change in Ntot, is 171.7947 with a standard error of 35.0191. The estimated slope is 171.7947. The lower limit of the 95% confidence interval for the slope is 101.0184 and the upper limit is 242.5710. The estimated intercept is −13.9802. The lower limit of the 95% confidence interval for the intercept is −28.2362 and the upper limit is 0.2758.

## 3. Discussion

Successful cultivation of quality potatoes is significantly influenced by the location with suitable soil and climatic conditions. The location significantly influences not only the yield itself but also the chemical composition of the potatoes [37,38,39,40]. Potatoes thrive on higher sites with higher rainfall and lower temperatures [39], with light soils. The negative impact of unfavourable climatic conditions (lower precipitation and higher temperatures) can be partly offset by the soil and its fertility. This is confirmed by our results, where the lowest average yields were recorded in Caslav (Table 1), a location with similar climatic conditions to Ivanovice, but with soil (degraded Chernozem) poorer in nutrients and low in soil carbon content (Table 2 and Table 3). In Ivanovice we can encounter similar climatic conditions as in Caslav, but the soil type here is Chernozem, a much more fertile soil compared to the degraded Chernozem found in Caslav. The difference in fertility (soil properties) between the two soils is due to the conditions of their formation [41]. The soil thus corrects the effect of climatic conditions (see Control results, Table 1), which resulted in higher yields than in Caslav. The highest yields were obtained in Lukavec, which offers the best natural conditions for potato cultivation. This finding is confirmed by PCA analysis (see Figure 2), where the Lukavec locality is significantly different (right upper corner) from the Ivanovice and Caslav localities. This is due to the Lukavec site having statistically significantly higher yields (on the PC2 axis of the component weights plot—significant correlation with Yield, r = −0.95), compared to the Caslav and Ivanovice sites. The Lukavec site is a typical potato production area compared to the Caslav and Ivanovice sites (maize production area). Here we can record average yields of 22 t ha^−1^ for the unfertilized Control (Table 1), which is also the average yield of early potatoes in the Czech Republic between the years 2015 and 2019 (the average yield of other potatoes in the Czech Republic was 28 t ha^−1^ between the years 2015 and 2019 [42]).

Weather conditions were the factor that most influenced potato yields at each of the three sites (see MANOVA results, Section 2.2.1, Section 2.2.2 and Section 2.2.3, first two lines), indicating potatoes sensitivity to weather and climate changes [43]. Both precipitation and temperature play an important role during the season and before the season’s start [28,43]. According to [44], night temperatures around 17 °C represent the optimum during the tuber formation process, while warmer temperatures significantly decrease the upcoming yields. The weather conditions not only affect the sizes and yields, but also the chemical composition of the potatoes [45]. When potatoes are subjected to stress conditions, whether caused by temperature, precipitation or a combination of these, potatoes respond with lower plant size, lower leaf area and cell membrane stability [46], resulting in lower yields. Such cumulative stress conditions were particularly evident in Caslav and Ivanovice in 2018, a year with exceptionally low yields (Table 1). It was the year characterized by very low precipitation and very high temperatures (Tables S1 and S2). Together with generally less suitable soil and climatic conditions, this occurrence of abnormal weather conditions resulted in average yields of 7.7 t ha^−1^ (Caslav) and 12.0 t ha^−1^ (Ivanovice). In Lukavec, we have recorded particularly low yields in 2018 (30.2 t ha^−1^) and especially in 2019 (26.3 t ha^−1^). The dry and warm start of the season in 2018 (Tables S1 and S2) slowed down the initial development of potatoes, but good precipitation in the following two months compensated such situation. In 2019, however, there was a spike in temperatures in May and June, when a very cold May was followed by an extraordinary warm June (Tables S1 and S2). We believe that it was this particular rapid development, coupled with the two extremes, that caused the unusually low yields in 2019, and the relatively normal conditions in the following months of the season did not help to restore the damages.

Fertilization was the second important factor that significantly affected potato yields at all locations (Table 1). Fertilization also affected soil properties (Table 2 and Table 3). In recent times, when livestock and crop production in the Czech Republic was in balance, potatoes represented (together with sugar beet) a crop traditionally fertilized with farmyard manure (FYM). Nowadays, this balance is disturbed as many companies do not keep livestock and their crop production strongly depends only on nitrogen from mineral fertilizers [17,47]. The combination of FYM and potatoes was (and still is, if FYM is available) a win-win solution, making potatoes an excellent pre–crop because manure positively modifies soil properties [31,48,49] and slowly releases all macronutrients [10], especially P, K and S [50]. The positive effect of FYM on crop yields and soil properties is well summarized in this meta-analysis [3]. According to [51], the recovery rate of K from FYM by crops vary between 24–26%. In our case, FYM application was always associated with higher yields (compared to unfertilized Control, Table 1). Statistically significant differences were not observed in each year, but over the entire study period, FYM provided significantly higher yields at each site. However, FYM application did not provide enough nutrients (N) to fulfil the yield potential of potatoes, especially in Caslav and Lukavec. The demonstrated dependence (linear regression model) of Ntot content in soil on yield is also related to N application to soil, including N mineralization in soil (Figure 4, r = 0.6129, R^2^ = 0.3756). Our obtained Pearson correlations (r) is higher compared to the study of authors [52] who considered r = 0.16 between crop yield in Premslin near Rostock in Germany. The process of mineralization (release of nutrients from FYM into the soil) strongly depends on soil and climatic conditions and can be strongly inhibited in the presence of inconvenient conditions, such as lack of precipitation. Therefore, the addition of mineral N significantly increased potato yields, especially at less fertile sites (Caslav, Lukavec). The FYM + NPK combinations significantly increased yields compared to the FYM + N treatments, again, especially in Caslav and Lukavec. This was confirmed by PCA and FA analysis (see Figure 2 and Figure 3), with the FYM + NPK treatments significantly higher within each subcluster on the PCA axis compared to Control and FYM + N. In Ivanovice, the differences were not so pronounced; the response of the potatoes to the fertilizer supplied was not so strictly noticeable due to the naturally fertile soil. In all cases, the difference between 80 (N2) and 120 (N3) kg N ha^−1^ was not statistically significant and 80 kg N ha^−1^ was sufficient to achieve good (reasonable) yields. The application of mineral P and K fertilizers covered the needs of potatoes to fulfil their potential, especially in less fertile soils (the difference in FYM + N treatments is significant in Caslav and Lukavec, Table 1) and left enough nutrients in the soil for the upcoming crop. This option represents the optimal form of fertilization to achieve high yields and ensure soil fertility. From the soil point of view, it is interesting that we did not observe any difference in pH values (Table 3). In conditions where only N fertilizers are applied to the soil, without the addition of organic matter (or with the addition of small doses of organic matter), the soil becomes acidic [15,21,22,53]. The application of FYM thus reduces the negative effects of mineral N fertilizers on soil pH [8,54]. The addition of FYM and FYM together with mineral fertilizers also increases soil carbon and nitrogen content (Table 3) [55], although the differences were not statistically significant everywhere. This is confirmed by the PCA analysis (Figure 2 and Figure 3), where the highest SOC and Ntot. the content was always recorded in FYM + NPK and FYM treatments, respectively, in all three localities (Caslav, Ivanovice, Lukavec, the FYM + NPK treatments are always significantly separated within each cluster–location, the FYM treatment is always second in order following the FYM + NPK treatment within PC1) compared to the Control treatment (lowest Cox and Ntot contents). Related to this finding is the demonstrated dependence (linear regression model) of content between Cox and Ntot in soil (Figure 3, r = 0.8675, R^2^ = 0.7526). From this point of view, the combined application of FYM with mineral NPK represents the optimal form of fertilization to achieve high yields and ensure soil fertility.

## 4. Materials and Methods

### 4.1. General Experiment Description

In 1955, three long–term field experiments were established to study the effect of twelve different fertilizer treatments and three soil–climate conditions on yield and quality parameters of arable crops and soil properties. According to Köpper—Geiger climate classification [56], all three sites are located in warm—summer humid continental climate (Dfb). The locations are Caslav (263 m a.s.l., 49°85′ N, 15°40′ E, soil type—calcic degraded Chernozems, arable layer: 40–45 cm), Ivanovice (225 m a.s.l., 49°19′ N, 17°05′ E, soil type—leptic Chernozems, arable layer: 30–35 cm) and Lukavec (620 m a.s.l., 49°57′ N, 14°99′ E, soil type—skeletic Cambisols, arable layer: 25–30 cm). Basic soil properties according to the fertilizer treatment in 2015 are shown in Table 5.

The weather conditions in each year (2016–2019), including a comparison with the standard climatological normal (1981–2010), are shown in Table 6. The specific precipitation and average temperatures in each month of the 2016–2019 seasons, including their comparison with the standard climatological normal (1981–2010), are shown in Supplementary Tables S1 and S2. The verbal assessment of the years (Table 6), months and growing seasons (Tables S1 and S2) were done according to [57].

The long-term field trials in Caslav, Ivanovice and Lukavec are uniform, so they have the same methodology. There are a total of four fields at each site (Field I, Field II, Field III, Field IV). Each of the four fields is divided into 48 plots of 8 by 8 m. A total of 12 different fertilizer treatments are applied to these plots, each treatment is repeated four times (12 × 4 = 48) in a completely randomized block design. From the total area of the single plot (8 m × 8 m), the central area of 5 m × 5 m is sampled for analyses (elimination of the edge effect).

In this paper, we evaluate four consecutive seasons (2016, 2017, 2018 and 2019) when potatoes (cul. Adéla) were grown. Planting (4800 kg ha^−1^) was always done in April and harvesting in September. The interline distance was 75 cm. Winter wheat was the pre–crop every year. For a better idea about the experimental design, please take a look at Table 7.

For this article, we have selected seven fertilization treatments out of a total of twelve: (1) unfertilized Control (unfertilized since the trial establishment), (2) the cattle farmyard manure (FYM), (3) and (4) FYM applied together with mineral N fertilizers (FYM + N1; FYM + N2), (5), (6) and (7) FYM applied together with mineral N, P and K fertilizers (FYM + NPK1; FYM + NPK2; FYM + NPK3. The specific fertilizer doses in each treatment are shown in Table 8. The rate indicated for mineral fertilizers represents the dosage of net nutrients applied to the field. FYM was applied at the dose of 40 t ha^−1^. The estimated nutrient content of the FYM is 200, 56 and 236 kg of N, P and K ha^−1^, respectively.

The mineral N was applied as calcium ammonium nitrate, P as triple superphosphate and K as potassium chloride. The wheat (pre–crop) harvest was followed by moderate stubble tillage. Subsequently, manure was applied to the field in autumn and incorporated into the soil by moderate tillage. The mineral fertilizers N1 (40 kg ha^−1^), N2 (80 kg ha^−1^), N3 (80 kg ha^−1^), P and K were applied during the pre-planting preparation in spring. The remaining 40 kg ha^−1^ N in the N3 treatment (together 120 kg ha^−1^, Table 8) was applied at the BBCH 16 stage. The FYM and mineral fertilizers were applied manually to the plots.

### 4.2. Soil Analyses

Following the potatoes harvest, soil samples were taken using the soil probes. The soil samples were taken from the topsoil layer (Caslav and Ivanovice 0–20 cm; Lukavec 0–15 cm). Four samples were taken from each plot. The samples were then mixed and transported to the laboratory for analysis. There, soil samples were dried and sieved. The value of the soil reaction (pH) was determined potentiometrically in 50 mL of 0.2 mol KCl (inoLab pH 730, WTW, Xylem Analytics, Weilheim, Germany). The SOC was determined colourimetrically and by oxidimetric titration according to [58,59]. The soil N content was determined with concentrated sulfuric acid in a heating block (Tecator, Foss A/S, Hillerød, Denmark), followed by the Kjeldahl method [60]. The concentrations of P, K, Mg and Ca were analyzed using the Mehlich III method [61], followed by the ICP—OES analysis (Thermo Scientific ICAP 7400 Duo, ThermoFisher Scientific, Cambridge, UK).

### 4.3. Data Analyses

For the evaluation of collected data, analysis of variance (ANOVA) and multivariate analysis of variance (MANOVA) were used using Statistica 13.3 (Tibco Software Inc., Palo Alto, California, USA). In the case of finding the significant differences, Tukey’s HSD post hoc analysis was performed. For the evaluation of the relationships between the yields and soil parameters, PCA (principal component analysis) and FA (factor analysis) were used (Statistica 14.0). The linear regression analyses were performed using the QC Expert 3.3 Pro (TriloByte Statistical Software Ltd., Pardubice, the Czech Republic) and NCSS 2019 Statistical Software (NCSS, LLC., Kaysville, UT, USA). The linear regression modelling used the regression triplet, consisting of (1) model design, (2) preliminary data analysis (multicollinearity, heteroskedasticity, autocorrelation and influence points), (3) estimation of parameters using the least square method (LSM) and subsequent testing of the significance of the parameter using the Student’s *t*-test, mean square error of prediction, and Akaike information criterion (AIC), (4) regression diagnostics—identification of influence points and verification of the LSM assumptions, (5) construction of the refined model [36]. Statistical significance was tested at a significance level of *p* = 0.05. The weather conditions were analyzed using MS Excel 2007 (Microsoft Corporation, Washington, DC, USA). The analyses, calculations and verbal evaluations were done according to [57], providing the recommendation of the World Meteorological Organization for a description of meteorological or climatological conditions. The weather data were collected from the weather stations running in the nearest vicinity of the field trials and operated by the Czech Hydrometeorological Institute (Prague, Czech Republic).

## 5. Conclusions

Potato cultivation is significantly influenced by soil and climatic conditions, which primarily affect yields. Suitable soil and climate conditions (lighter soil, higher altitude, higher rainfall, lower temperatures—Lukavec) allow average yields to be achieved, even without the addition of mineral fertilizers. In less suitable conditions (heavier soils, higher temperatures, less rainfall—Caslav, Ivanovice), it depends on the fertility of the soil (soil type) whether it can compensate for the climate deficiencies. The temperature was the parameter strongly and negatively affecting potato yields in our trial (more than precipitation). The occurrence of extraordinary temperatures (Table S2) significantly reduced potato yield, especially in 2018 at all locations. These yield fluctuations (the effect of weather on yields) are and will be encountered more frequently as the occurrence of such affected seasons is predicted to be more frequent.

Manure is a form of fertilizer that significantly increases potato yields, which is an important fact, especially for organic farming. However, without the addition of mineral fertilizers, the modern potato cultivars grown under conventional agriculture practices cannot fully fulfil their yield potential as their requirements for nutrients are higher. The application of manure together with mineral forms of NPK ensures high yields. A dose of 80 kg N ha^−1^ gave comparable yields to a dose of 120 kg N ha^−1^ and represents a reasonable dose in terms of price/performance ratio.

Application of FYM and especially FYM + NPK significantly increased the soil P and K concentrations in Ivanovice and Lukavec, leaving sufficient nutrient reserves in the soil for the upcoming crop. Manure application also slightly (statistically insignificantly) increased the soil pH at all sites but mainly prevents the negative effect of nitrogen fertilizers on lowering the soil pH, which is important information for agriculture that is significantly dependent on mineral nitrogen fertilizers and has long been struggling with a lack of organic manures applied to the soil, as is the case of the Czech Republic.

## Figures and Tables

**Table 1:** The effect of the fertilizer treatments on potato yields as affected by the year (2016–2019) and locality (Caslav, Ivanovice, Lukavec).

	Control	FYM	FYM + N1	FYM + N2	FYM + NPK1	FYM + NPK2	FYM + NPK3	Mean
**Caslav**								
2016	8.6 ± 0.4 a	14.9 ± 1.1 b	20.0 ± 0.9 c	19.9 ± 0.2 c	25.4 ± 1.2 d	32.7 ± 1.2 e	30.0 ± 0.8 e	21.7 ± 1.5 D
2017	7.1 ± 0.7 a	9.3 ± 0.7 ab	10.3 ± 0.3 ab	12.3 ± 0.6 bc	16.2 ± 1.1 cd	19.9 ± 1.0 d	25.3 ± 2.0 e	14.3 ± 1.2 B
2018	4.4 ± 0.2 a	6.6 ± 0.3 a	7.5 ± 0.2 ab	7.9 ± 0.3 abc	8.6 ± 0.6 abc	9.5 ± 0.7 c	9.3 ± 0.4 bc	7.7 ± 0.3 A
2019	10.6 ± 0.4 a	11.8 ± 0.3 b	16.7 ± 0.5 c	20.8 ± 0.7 d	21.3 ± 0.4 de	23.2 ± 0.8 de	23.6 ± 0.6 e	18.3 ± 1.0 C
Mean	7.7 ± 0.6 a	10.6 ± 0.9 b	13.6 ± 1.3 c	15.2 ± 1.4 c	17.9 ± 1.7 d	21.3 ± 2.2 e	22.1 ± 2.1 e	
**Ivanovice**								
2016	18.6 ± 0.2 a	28.7 ± 0.9 b	30.5 ± 0.8 bc	31.9 ± 0.8 bc	30.9 ± 1.2 bc	34.0 ± 2.2 bc	35.1 ± 2.0 c	29.9 ± 1.1 C
2017	15.1 ± 2.1 a	20.3 ± 1.7 ab	20.7 ± 0.5 ab	23.4 ± 0.9 b	24.4 ± 0.6 b	25.1 ± 1.6 b	23.8 ± 1.3 b	21.8 ± 0.8 B
2018	8.9 ± 1.4 a	11.9 ± 2.6 a	11.0 ± 2.0 a	11.6 ± 2.9 a	12.5 ± 1.9 a	14.2 ± 2.8 a	13.7 ± 0.8 a	12.0 ± 0.8 A
2019	17.2 ± 0.6 a	26.8 ± 1.6 b	32.1 ± 2.2 bc	28.8 ± 1.2 b	33.9 ± 2.5 bc	34.9 ± 2.5 bc	38.3 ± 1.1 c	30.3 ± 1.4 C
Mean	15.0 ± 1.1 a	21.9 ± 1.9 b	23.6 ± 2.3 bc	23.9 ± 2.1 bc	25.4 ± 2.3 bcd	27.1 ± 2.4 cd	27.7 ± 2.6 d	
**Lukavec**								
2016	20.4 ± 1.8 a	29.1 ± 2.2 a	41.9 ± 2.3 b	48.9 ± 3.7 bc	43.7 ± 2.6 b	49.4 ± 2.9 bc	59.7 ± 1.3 c	41.9 ± 2.5 C
2017	29.6 ± 2.6 a	41.9 ± 1.9 b	45.1 ± 2.0 b	49.6 ± 2.7 bc	59.1 ± 0.9 cd	64.4 ± 0.9 c	64.7 ± 2.8 c	50.6 ± 2.4 D
2018	22.9 ± 1.9 a	29.0 ± 0.4 ab	24.4 ± 0.6 a	26.7 ± 0.7 ab	34.0 ± 1.4 bc	36.6 ± 3.4 c	37.7 ± 0.8 c	30.2 ± 1.2 B
2019	15.1 ± 0.5 a	19.2 ± 0.8 a	25.6 ± 0.5 b	31.3 ± 1.0 c	32.4 ± 1.7 c	30.6 ± 1.5 bc	29.7 ± 1.2 bc	26.3 ± 1.2 A
Mean	22.0 ± 1.6 a	29.8 ± 2.2 b	34.2 ± 2.5 c	39.1 ± 2.9 d	42.3 ± 2.9 de	45.2 ± 3.5 ef	47.9 ± 3.9 f	

The mean values (±standard error) followed by the same letter (a—vertically—comparing the fertilizer treatments, A—horizontally—comparing the years in individual localities) are not statistically different (*p* < 0.05).

**Table 2:** The long–term effect of the fertilization treatments on soil P, K, Mg and Ca (mg kg^−1^) concentrations.

	P	P Assess.	K	K Assess.	Mg	Mg Assess.	Ca
**Caslav**							
Control	49 ± 12 ^A^	Low	120 ± 14 ^A^	Suitable	145 ± 26 ^A^	Suitable	2888 ± 118 ^A^
FYM	46 ± 6 ^A^	Low	135 ± 16 ^A^	Suitable	136 ± 4 ^A^	Suitable	3777 ± 570 ^A^
FYM + N2	55 ± 11 ^A^	Suitable	141 ± 11 ^A^	Suitable	156 ± 6 ^A^	Suitable	2802 ± 94 ^A^
FYM + NPK2	158 ± 9 ^B^	High	221 ± 21 ^B^	Good	164 ± 3 ^A^	Good	2950 ± 198 ^A^
**Ivanovice**							
Control	66 ± 10 ^A^	Suitable	181 ± 6 ^A^	Good	204 ± 17 ^A^	Good	4102 ± 152 ^A^
FYM	169 ± 15 ^C^	High	370 ± 27 ^C^	High	234 ± 10 ^A^	Good	4131 ± 166 ^A^
FYM + N2	117 ± 12 ^B^	High	288 ± 21 ^B^	Good	252 ± 14 ^A^	Good	4232 ± 152 ^A^
FYM + NPK2	226 ± 8 ^D^	Very high	447 ± 17 ^C^	Very high	236 ± 5 ^A^	Good	4150 ± 226 ^A^
**Lukavec**							
Control	44 ± 1 ^A^	Low	107 ± 6 ^A^	Suitable	109 ± 9 ^A^	Suitable	2050 ± 82 ^A^
FYM	90 ± 8 ^B^	Good	147 ± 8 ^BC^	Suitable	126 ± 7 ^A^	Suitable	2125 ± 96 ^A^
FYM + N2	46 ± 3 ^A^	Low	123 ± 5 ^AB^	Suitable	113 ± 9 ^A^	Suitable	2214 ± 124 ^A^
FYM + NPK2	164 ± 5 ^C^	High	167 ± 4 ^C^	Suitable	104 ± 9 ^A^	Low	2182 ± 98 ^A^

The mean values (±standard error) followed by the same letter (^A^—vertically—comparing the fertilizer treatments) are not statistically different (*p* < 0.05).

**Table 3:** The long–term effect of fertilization on the value of soil pH, Cox (%) and Ntot (%) content.

	pH (KCl)	C_ox_ (%)	N_tot_
**Caslav**			
Control	6.57 ± 0.03 ^A^	1.17 ± 0.06 ^A^	0.15 ± 0.01 ^A^
FYM	6.85 ± 0.14 ^A^	1.20 ± 0.05 ^A^	0.15 ± 0.01 ^A^
FYM + N2	6.51 ± 0.07 ^A^	1.20 ± 0.05 ^A^	0.16 ± 0.01 ^A^
FYM + NPK2	6.53 ± 0.17 ^A^	1.29 ± 0.09 ^A^	0.17 ± 0.01 ^A^
**Ivanovice**			
Control	6.58 ± 0.14 ^A^	1.67 ± 0.03 ^A^	0.20 ± 0.01 ^A^
FYM	6.69 ± 0.08 ^A^	1.92 ± 0.06 ^B^	0.23 ± 0.01 ^A^
FYM + N2	6.62 ± 0.14 ^A^	1.95 ± 0.03 ^B^	0.23 ± 0.01 ^A^
FYM + NPK2	6.63 ± 0.11 ^A^	2.07 ± 0.05 ^B^	0.24 ± 0.01 ^A^
**Lukavec**			
Control	5.84 ± 0.06 ^A^	1.41 ± 0.05 ^A^	0.19 ± 0.01 ^A^
FYM	5.88 ± 0.12 ^A^	1.72 ± 0.06 ^AB^	0.23 ± 0.01 ^B^
FYM + N2	5.74 ± 0.08 ^A^	1.72 ± 0.03 ^AB^	0.23 ± 0.01 ^B^
FYM + NPK2	5.83 ± 0.05 ^A^	1.82 ± 0.13 ^B^	0.23 ± 0.01 ^B^

The mean values (±standard error) followed by the same letter (^A^—vertically—comparing the fertilizer treatments) are not statistically different (*p* < 0.05).

**Table 4:** Factor weights and contributions of given factors to the communality for individual characters after normalized Varimax rotation for production (yield) and soil parameters.

Variable	Factor Weights		Contribution of Factors		
	Factor 1	Factor 2	Factor 1	Factor 2	Communality
pH (KCl)	−0.2978	0.9357	0.0887	0.9644	0.9876
P	0.7492	0.3972	0.5614	0.7192	0.9473
K	0.6569	0.7101	0.4315	0.9359	0.9607
Ca	0.1134	0.9560	0.0128	0.9268	0.9914
Mg	0.3480	0.8936	0.1211	0.9196	0.9397
Cox	0.9498	0.1717	0.9022	0.9317	0.9983
Ntot	0.9693	−0.0704	0.9396	0.9445	0.9977
Yield	0.7811	−0.5738	0.6101	0.9394	0.9336

**Table 5:** Soil pH, the concentration of P, K, Ca and Mg (mg kg^−1^) and contents of organic carbon (Cox, %) and total nitrogen (Nt, %) in Caslav, Ivanovice and Lukavec in 2015 (the season before the evaluated period).

	pH	P	K	Ca	Mg	Cox	Nt
**Caslav**							
Control	6.72	58	114	2858	99	1.26	0.14
FYM	6.55	58	144	2891	118	1.16	0.13
FYM + N2	6.76	76	125	2891	139	1.31	0.16
FYM + NPK2	6.52	188	225	2802	122	1.43	0.17
**Ivanovice**							
Control	6.85	101	228	4451	200	1.72	0.19
FYM	6.84	171	340	4371	222	1.99	0.23
FYM + N2	6.86	156	320	4481	238	2.13	0.25
FYM + NPK2	6.82	220	438	4215	223	2.04	0.23
**Lukavec**							
Control	5.93	40	131	1945	92	1.54	0.20
FYM	5.93	56	157	2096	108	1.82	0.23
FYM + N2	5.78	38	164	2047	99	1.86	0.23
FYM + NPK2	5.89	193	207	2011	96	1.82	0.23

**Table 6:** The annual sum of precipitation (mm) and the annual mean temperature (°C) compared with the standard climatological normal (1981–2010) in Caslav, Ivanovice and Lukavec (2016–2019).

	Precipitation	Evaluation	Temperature	Evaluation
Caslav				
Normal	593		9.4	
2016	393	V. B. Normal ^4^	9.7	Normal
2017	633	Normal	9.6	Normal
2018	318	E. B. Normal ^6^	10.8	V. A. Normal ^2^
2019	478	B. Normal ^4^	10.6	A. Normal ^1^
Ivanovice				
Normal	562		9.1	
2016	474	B. Normal ^4^	9.9	A. Normal ^1^
2017	411	V. B. Normal ^5^	9.8	A. Normal ^1^
2018	384	V. B. Normal ^5^	11.0	E. A. Normal ^3^
2019	740	V. A. Normal ^2^	10.8	E. A. Normal ^3^
Lukavec				
Normal	698		7.8	
2016	601	B. Normal ^4^	7.9	Normal
2017	777	A. Normal ^1^	7.9	Normal
2018	509	V. B. Normal ^5^	8.8	A. Normal ^1^
2019	680	Normal	9.1	V. A. Normal ^2^

Note: ^1^ Above Normal; ^2^ Very Above Normal; ^3^ Extraordinary Above Normal; ^4^ Below Normal; ^5^ Very Below Normal; ^6^ Extraordinary Below Normal.

**Table 7:** The scheme of the trial in the period 2015–2019.

	Field I.	Field II.	Field III.	Field IV.
2015	Winter wheat			
2016	Potatoes	Winter wheat		
2017		Potatoes	Winter wheat	
2018			Potatoes	Winter wheat
2019				Potatoes

**Table 8:** The doses of the nutrients applied in the FYM and individual fertilizer treatments (kg ha^−1^).

Fertilization Treatment Designation	N	P	K
Control	0	0	0
FYM (40 t ha^−1^)	200	56	236
FYM + N1 (kg ha^−1^)	240	56	236
FYM + N2 (kg ha^−1^)	280	56	236
FYM + NPK1	240	136	336
FYM + NPK2	280	136	336
FYM + NPK3	320	136	336

## Data Availability

We would like to exclude this statement as the study does not report any online available data.

## Supplementary Materials

The following are available online at https://www.mdpi.com/article/10.3390/plants10112473/s1, Table S1: The average monthly sum of precipitation (mm) in Caslav, Ivanovice and Lukavec in comparison with the climate normal (1981–2010), Table S2: The average monthly temperatures (°C) in Caslav, Ivanovice and Lukavec in comparison with the climate normal (1981–2010).
