# Supplementary material for: The Effect of Soil-Climate Conditions, Farmyard Manure and Mineral Fertilizers on Potato Yield and Soil Chemical Parameters
Source: Plants (Basel). 2021 Nov 16;10(11):2473. doi: 10.3390/plants10112473 (PMC8625073; doi:10.3390/plants10112473)
Supplement: Supplementary file 1 [file plants-10-02473-s001.zip › plants-1417679-supplementary.pdf]

Table S1. The average monthly sum of precipitation (mm) in Caslav, Ivanovice and Lukavec in comparison with the climate normal (1981 – 2010).

	April	May	June	July	August	September	Sum
**Caslav**							
Normal	35.6	68.8	67.7	92.9	78.5	54.2	397.7
2016	19.4	35.2	69.0	84.8	33.7	8.1	250.2
2017	86.9	39.5	80.0	100.8	97.3	48.6	453.1
2018	13.0	33.6	47.3	15.2	24.2	36.3	169.6
2019	26.3	85.0	23.4	65.6	65.1	39.4	304.8
**Ivanovice**							
Normal	31.9	67.8	75.7	71.3	65.6	52.0	364.3
2016	48.9	36.6	28.0	108.8	42.7	14.3	279.3
2017	43.2	25.6	41.6	73.9	35.1	72.2	291.6
2018	20.0	27.2	52.0	42.6	21.4	65.3	228.5
2019	18.2	85.5	186.9	84.1	85.4	79.1	539.2
**Lukavec**							
Normal	45.1	63.7	78.5	93.4	88.1	58.3	427.0
2016	32.0	89.3	58.4	110.5	22.8	16.4	329.4
2017	102.1	33.0	66.0	135.4	67.1	35.0	438.6
2018	7.2	51.5	65.7	43.6	38.9	78.3	285.2
2019	16.8	96.6	39.2	94.0	62.2	35.9	344.7

Above normal	Very above normal	Extraordinary above normal
Below normal	Very below normal	Extraordinary below normal

Table S2. The average monthly temperatures (°C) in Caslav, Ivanovice and Lukavec in comparison with the climate normal (1981 – 2010).

	April	May	June	July	August	September	Mean
**Caslav**							
Normal	9.4	14.6	17.3	19.3	18.8	14.4	15.7
2016	8.5	14.2	17.8	19.4	17.8	16.8	15.8
2017	8.4	14.8	18.5	19.2	19.4	12.5	15.5
2018	13.3	17.1	18.6	20.9	21.9	15.2	17.8
2019	10.2	11.9	21.4	19.5	19.8	14.3	16.2
**Ivanovice**							
Normal	9.6	14.8	17.6	19.7	19.2	14.4	15.9
2016	8.9	14.7	19.0	20.3	18.9	17.1	16.5
2017	8.6	15.2	20.2	20.6	21.3	13.8	16.6
2018	14.5	18.0	19.5	21.5	23.0	16.3	18.8
2019	11.1	12.4	22.0	20.0	20.9	15.0	16.9
**Lukavec**							
Normal	7.6	12.9	15.6	17.6	17.1	12.7	13.9
2016	6.9	12.2	16.3	17.9	16.6	15.3	14.2
2017	5.8	13.0	17.4	17.8	18.5	11.0	13.9
2018	12.2	15.0	16.5	18.7	20.3	14.2	16.2
2019	8.1	9.5	20.1	17.4	18.5	13.3	14.5

Above normal	Very above normal	Extraordinary above normal
Below normal	Very below normal	
